# Effects of magnetic monopoles charge on the cracking reversal processes in artificial square ices

**DOI:** 10.1038/s41598-020-66794-0

**Published:** 2020-06-19

**Authors:** T. S. de Paiva, J. H. Rodrigues, L. A. S. Mól, A. R. Pereira, J. Borme, P. P. Freitas, C. I. L. de Araujo

**Affiliations:** 1 Laboratory of Spintronics and Nanomagnetism (LabSpiN), Departamento de Física, Universidade Federal de Viçosa, Viçosa, 36570-900 Minas Gerais Brazil; 2 Nucleo de Física, Instituto Federal de Minas Gerais - Campus Bambuí BR, 38900-000 Bambuí, Minas Gerais Brazil; 3 Laboratorio de Simulação, Departamento de Física, ICEx, Universidade Federal de Minas Gerais, 31720-901 Belo Horizonte, Minas Gerais Brazil; 4 INL-International Iberian Nanotechnology Laboratory, 4715-330 Braga, Portugal

**Keywords:** Magnetic devices, Magnetic properties and materials

## Abstract

In this paper we perform nanofabrication of square artificial spin ices with different lattice parameters, in order to investigate the roles of vertex excitation on the features of the system. In particular, the character of magnetic charge distribution asymmetry on the vertices are observed under magnetic hysteresis loop experiments. We then compare our results with simulation using an emergent Hamiltonian containing objects such as magnetic monopoles and dipoles in the vertices of the array (instead of the usual Hamiltonian based on the dipolar interactions among the magnetic nanoislands). All possible interactions between these objects are considered (monopole-monopole, monopole-dipole and dipole-dipole). Using realistic parameters we observe very good match between experiments and theory, which allow us to better understand the system dynamics in function of monopole charge intensity.

## Introduction

**Figure 1:**
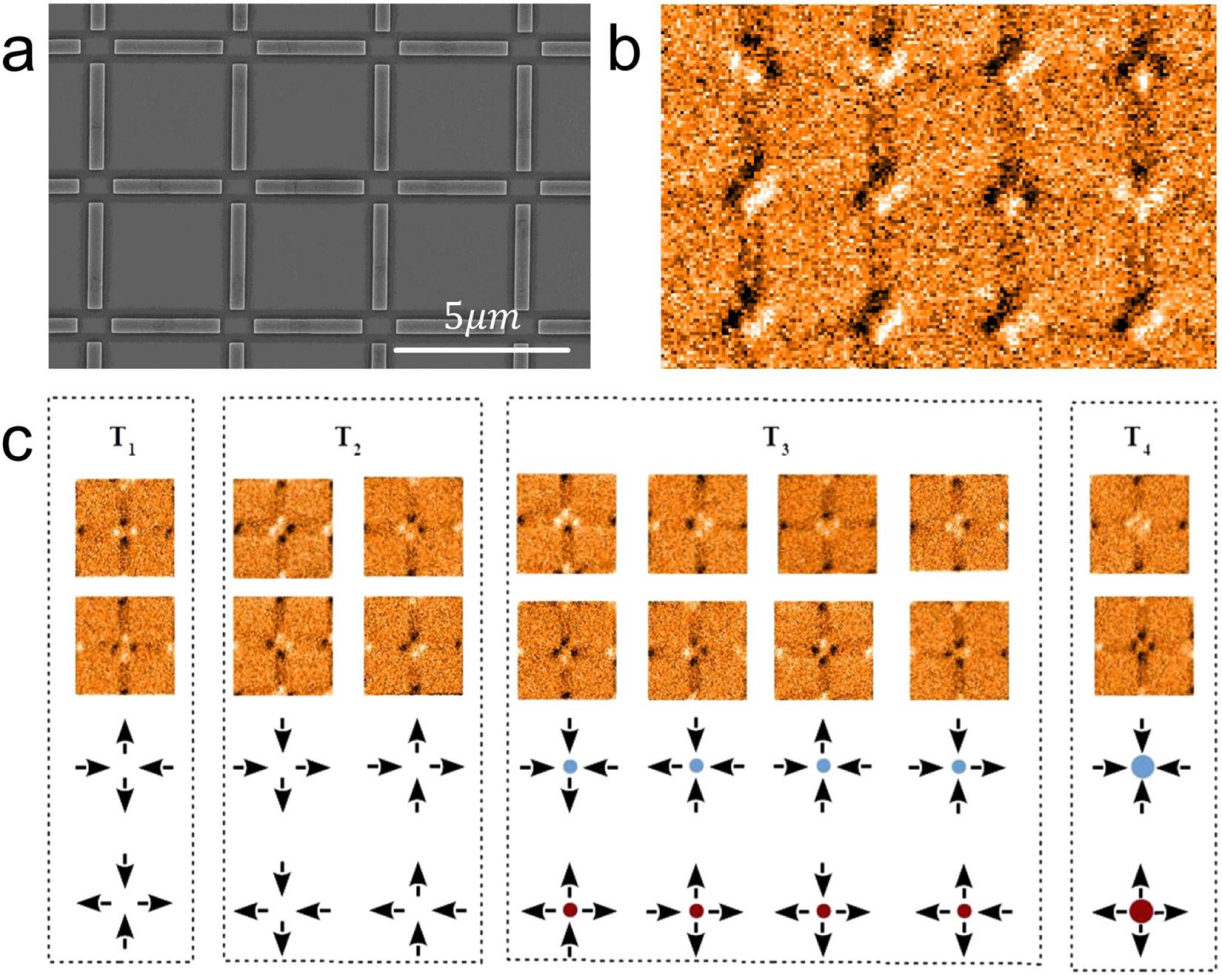
(**a**) Artificial spin ice in a square lattice measured by *SEM*. (**b**) magnetization of nanoislands in same array characterized by *MFM* (**c**) The four possible classes for the spin arrangements in a vertex *T*_1_, *T*_2_, *T*_3_ and *T*_4_.

Emergent phenomena are characterized by exhibiting new particles and fields which are completely absent in the original Hamiltonian that describes a system. For instance, such features can be seen in a variety of condensed matter materials (one-dimensional electronic fluids with the exotic spinons and holons^1,2^; natural spin ices with magnetic monopoles^3^ and many others). For artificial spin ices (*ASI*), built with elongated magnetic nanoislands (having a net Ising magnetic moment), the original Hamiltonian is essentially based on the dipolar interactions among the dipoles displayed in determined geometry, which frustrates the system (see Fig. 1), similar to what happens in the water ice. For the square array, this Hamiltonian is given, in general, by ${H}_{dip}=D{\sum }_{i > j}\,\left[\frac{{\hat{e}}_{i}\cdot {\hat{e}}_{j}}{{r}_{ij}^{2}}-\frac{3({\hat{e}}_{i}\cdot {\overrightarrow{r}}_{ij})({\hat{e}}_{j}\cdot {\overrightarrow{r}}_{ij})}{{r}_{ij}^{5}}\right]{s}_{i}{s}_{j}$, where *D* is the coupling constant of the dipolar interaction, ${\hat{e}}_{i}$ is the local Ising axes of the lattice, *r*_*ij*_ is the distance between magnetic moments (spins) and *s*_*i*_ = ±1 represents the two states (up/down for vertical islands and right/left for horizontal islands) of the Ising spin. The *ASI* system was firstly produced in 2006 in a square lattice by Wang *et. al*.^4^. Of course, this planar little world is constituted by the traditional objects of our universe (the nanoislands are obviously magnetic dipoles as explicitly seen in the above Hamiltonian), but their large number and interactions may produce the phenomenon of fractionalization (“more is different”^5^). Indeed, it was shown that such artificial spin ices support north and south magnetic monopole quasiparticles^6,7^ connected by energetic strings (a kind of Nambu monopoles^8–10^) above the ground state. These atypical objects, as well as the distinct monopoles present in the natural spin ices^3^, are emergent quasiparticles (coming from many dipoles in interaction) and their magnetic charges are not at all constrained (as usually occur with their quantum field theory counterparts). Here, the emergent Nambu monopoles are responsible for several features of the *ASI* materials The purpose of this paper is to study theoretically and experimentally an artificial square spin ice (ASSI) by considering an alternative Hamiltonian which contains only the emergent excitations (such as monopoles) as protagonists and not the original and concrete dipole nanoislands. This model was recently proposed to measure the excitations (monopole-like and dipole-like) interactions by Rodrigues and Mól^11^. In ref. ^11^, it was observed that the monopole density in a magnetization reversal process is affected by the charge of monopoles in such a way that, for increasing monopole charge, an increasing kurtosis and skewness in monopole distribution could be envisaged. In addition, one may expect that, for a larger lattice spacing, the effective monopole charge of a vertex would be reduced due to the smaller magnetic field density in that region. A signature of this effect would be an increase of the maximum monopole density for increasing lattice spacings. Thus, we have studied three different realizations of an ASSI, with different lattice spacings, searching for possible signatures of modifications of the monopoles charge. Our results suggest that localized modifications on monopoles charge or, equivalently, on the internal barrier for spin flips, are responsible for the existence of cracking reversal in the hysteresis curve. In what follows we describe some necessary background to understand our work, including the theoretical model; then we present the experimental and theoretical results. Finally our conclusions are exposed.

## Background

Before describing the theoretical model we briefly summarize some well known facts about the square lattice investigated here. The ground state of the artificial square ice obeys the famous ice rule, which remains the familiar two-in, two-out (two spins must point in, while the other two must point out in each vertex). Figure 1a presents Scanning Electron Microscopy (*SEM*) of a developed sample while Fig. 1b shows the magnetization response of nanoislands measured by Magnetic Force Microscopy (*MFM*).

From this measurements, it is possible to observe all possible vertex configurations separated by classes having the same energy, indicated by *T*_1_, *T*_2_, *T*_3_ and *T*_4_ (see Fig. 1c). The first two categories (*T*_1_ and *T*_2_) obey the ice rule but the energy of these states is not degenerate (vertex configurations *T*_1_ has smaller energy than the ones with configurations *T*_2_). Indeed, vertices *T*_2_ have a resulting dipole moment not present in vertices type *T*_1_. The other two categories (*T*_3_ and *T*_4_) are excited states usually associated with monopole like excitation^6,7,12–18^. Of course, the ground-state of this system requires all vertices to be category *T*_1_.

With this in mind, the Hamiltonian which describes an emergent vertex model can be elaborated to explore how modifications in the Coulomb interactions affect the distribution of monopole-like excitation in a magnetization reversal process. It is given by^11^

$${H}_{v}=\frac{{\mu }_{0}{q}^{2}}{4\pi a}\sum _{i < j}\frac{{Q}_{i}{Q}_{j}}{{r}_{ij}}-\frac{{\mu }_{0}{q}^{2}l}{4\pi {a}^{2}}\sum _{i < j}\frac{{\overrightarrow{p}}_{i}\cdot {\hat{r}}_{ij}}{{r}_{ij}^{2}}+\frac{{\mu }_{0}{q}^{2}{l}^{2}}{4\pi {a}^{3}}\sum _{i < j}[\frac{{\overrightarrow{p}}_{i}\cdot {\overrightarrow{p}}_{j}-\mathrm{3(}{\overrightarrow{p}}_{i}\cdot {\hat{r}}_{ij}\cdot {\overrightarrow{p}}_{j}\cdot {\hat{r}}_{ij})}{{r}_{ij}^{3}}]+{E}_{c}^{{T}_{2}}\sum _{i}{\delta }_{i,{T}_{2}}+{E}_{c}^{{T}_{3}}\sum _{i}{\delta }_{i,{T}_{3}}+{E}_{c}^{{T}_{4}}\sum _{i}{\delta }_{i,{T}_{4}},$$

where a dimensionless charge for vertex *i* assumes the values *Q*_*i*_ = 0 if vertex *i* is on category *T*_1_ or *T*_2_, *Q*_*i*_ = ±2 if it is on category *T*_3_ and *Q*_*i*_ = ±4 if it is on category *T*_4_. The dimensionless dipole moment of vertex *i* is $|{\overrightarrow{p}}_{i}|=0$ if it is on category *T*_1_ or *T*_4_; $|{\overrightarrow{p}}_{i}|=1$ if it is on category *T*_3_ and $|{\overrightarrow{p}}_{i}|=\sqrt{2}$ if it is on category *T*_2_. Also, ${E}_{c}^{{T}_{j}}$ is the creation energy of a vertex on category *T*_*j*_ (in units of *D*) and ${\delta }_{i,{T}_{j}}=1$ if vertex *i* is on category *T*_*j*_ and 0 otherwise; *q* is the magnitude of the magnetic charge (in units of *μ*/*a*) and *m* = *ql* is the magnitude of the dipole moment of a vertex. The constant *a* is the lattice spacing and *μ* is the nanoisland’s magnetic dipole moment. Therefore, this Hamiltonian considers the emergent monopole like quasiparticles and the resulting typical dipoles arising in categories *T*_2_ and *T*_3_. The interactions among the monopoles are given by the Coulomb potential (first term) while the second term represents the interactions among monopoles and dipoles and the third term indicates the dipole-dipole interactions. Finally. the last three terms represent the creation energy for each kind of vertex (emergent excitation); the creation energy of a vertex on category *T*_1_ was set to zero, in such a way that the ground-state energy is equal to zero. In ref. ^11^, there is a complete discussion about the values of the constants *q*, *l*, ${E}_{c}^{{T}_{2}}$, ${E}_{c}^{{T}_{3}}$, ${E}_{c}^{{T}_{4}}$, including also *ql*. There, they were obtained by using an equivalence between the point dipole model and the above emergent model. The best results for this equivalence suggest that the border configurations should be considered while the differences introduced by constraining *ql* to *μ* may lead only to small modifications, expected to be irrelevant in comparison to thermal energy or imperfections present in real systems. Here, we use the values adopted in ref. ^11^, where more details about the model can be found. We have to lay emphasis on the fact that the emergent model introduced here is somewhat related to several works, which deal with models of emergent monopoles and Coulomb phase, usually applied to study the monopole density during magnetization or thermal processes in square or pyrochlore lattices^17,19–25^.

## Results and Discussions

### Experiments

Samples were fabricated on silicon substrate from a previous multilayer prepared by sputtering technique with composition *Si*/*Ta* 3 *nm*/*Ni*_80_*Fe*_20_ · 10 *nm*/*Ta* 3 *nm*; tantalum was used as seed and cap layer. For the definition of the nanoislands a 85 *nm* layer of *AR*–*N*7520.18 negative tone electroresist was spin coated onto the multilayer and electron beam lithography was performed at 100 *kV* of acceleration voltage. After development, the samples were submitted to ion mill at 20° from normal incidence and the etching end was controlled by secondary ion mass spectroscopy detection. Finally, electroresist was removed from the top of nanoisland by ashing in oxygen plasma. As in ref. ^18^, the nanoislands dimensions of *l* = 2800 *nm* and *w* = 400 *nm* were chosen as limit size to present magnetic monodomain in each island with highest magnetization for good response in magnetic measurements. We have developed samples with three different lattice spacings of *SQ*_0_ = 3550 *nm*, *SQ*_4_ = 3950 *nm* and *SQ*_8_ = 4350 *nm* as an attempt to modify monopoles charge. The samples were characterized by Magnetic Force Microscopy (*MFM*) with an adapted external magnet. Images were taken after each step of external magnetic field application. From the images, the magnetization and vertex topology were mapped for each external magnetic field step.

**Figure 2:**
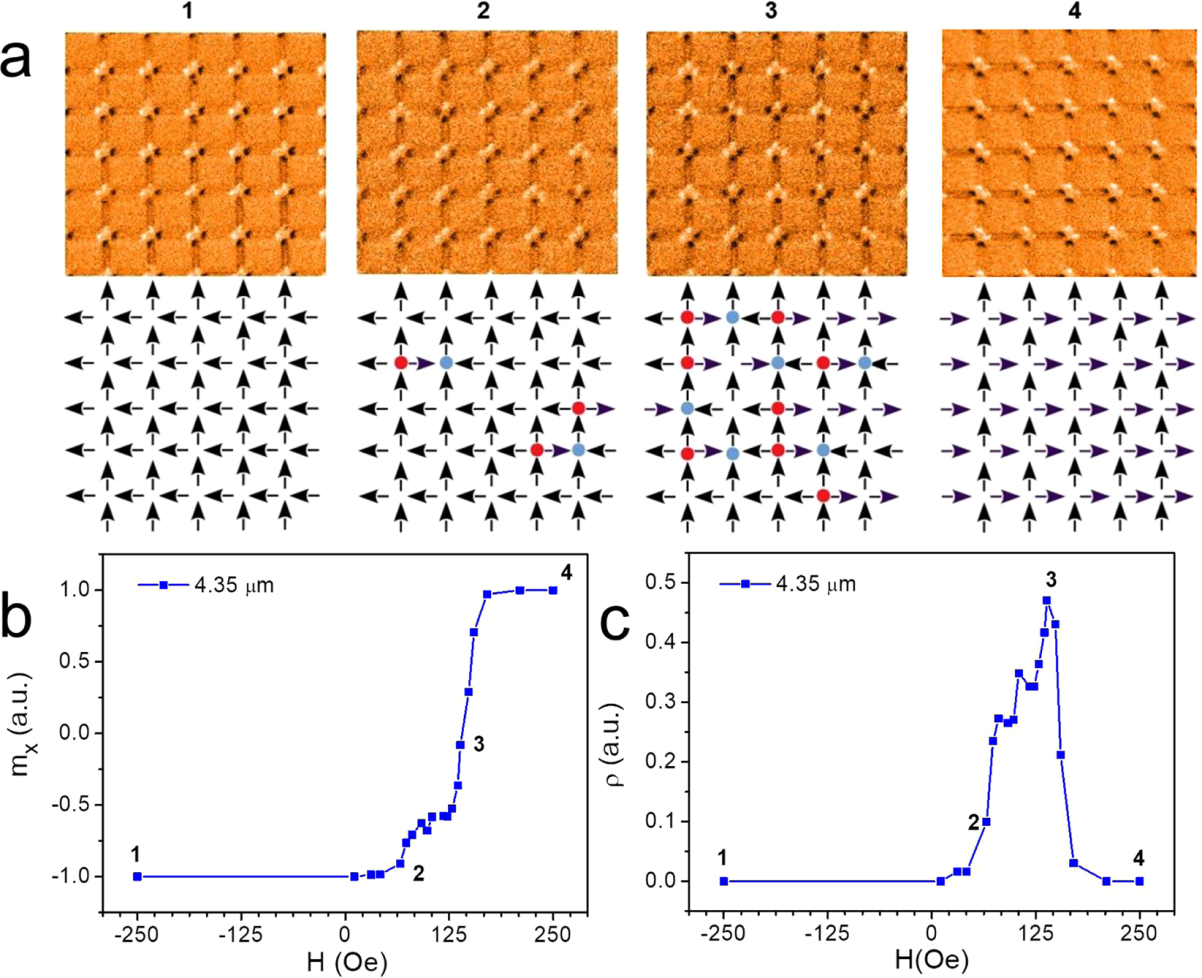
Experimental results for a square ice in the presence of a magnetic field applied along the *x*-direction. (**a**) Magnetic Force Microscopy (*MFM*) view and its schematics for the spin directions. The range of the external field *H* goes from −250Oe to +250Oe. Points 1, 2, 3 and 4 illustrate four different configurations for the spins in the square *ASI* as the field varies. Parts (**b**) and (**c**) of the figure show a comparison among the spin configurations corresponding to these points with the behavior of the magnetization *m*_*x*_ and monopole density *ρ* (as a function of *H*) respectively.

We now describe our experimental results in order to compare with the emergent model expressed by *H*_*v*_. Figure 2 shows how excitations are created as an external magnetic field is applied. In particular, Fig. 2a displays the configurations of the array for 4 specific values of the field. The behavior of both, the *x*-component of the magnetization (*m*_*x*_) and vertex population *ρ*, as a function of the applied magnetic field along the *x*-direction are shown in Fig. 2b,c respectively. The range of the external field goes from *H* = −250Oe to *H* = +250Oe. The system remains with a magnetization *m*_*x*_ = −1 (in arbitrary unit), keeping a complete state of saturation as the field varies from *H* = −250*Oe* to zero, when it will start to change. Meanwhile, the density of magnetic charges also starts to increase as the field becomes positive, reaching a maximum value at *H* ~ 125 Oe, when it starts to decrease to zero again as the magnetization reaches the saturation (*m*_*x*_ = +1). The points 1, 2, 3 and 4 in Fig. 2 show typical configurations for the spins in the square ice during this process. By passing from negative to positive magnetization *m*_*x*_, some magnetic charges are created, in general in pairs (monopole-antimonopole), but the lattice borders affect this distribution as will be discussed below. It is interesting to observe how the magnetization increases as the field increases from zero, mainly in the field range between *H* ~ 70 Oe and *H* ~ 125 Oe where *ρ* grows to a maximum. Out of this range, the magnetization behaves as expected and after *H* ~ 125*Oe* there is an accentuated reduction of *ρ* and the magnetic charges rapidly disappear from the system. Therefore, the monopoles may be responsible for a kind of cracking reversal behavior of the magnetization when the field varies in the interval (70Oe–125Oe). The emergent theory (*H*_*v*_), which treats directly with the magnetic charges, will help us to understand this effect.

**Figure 3:**
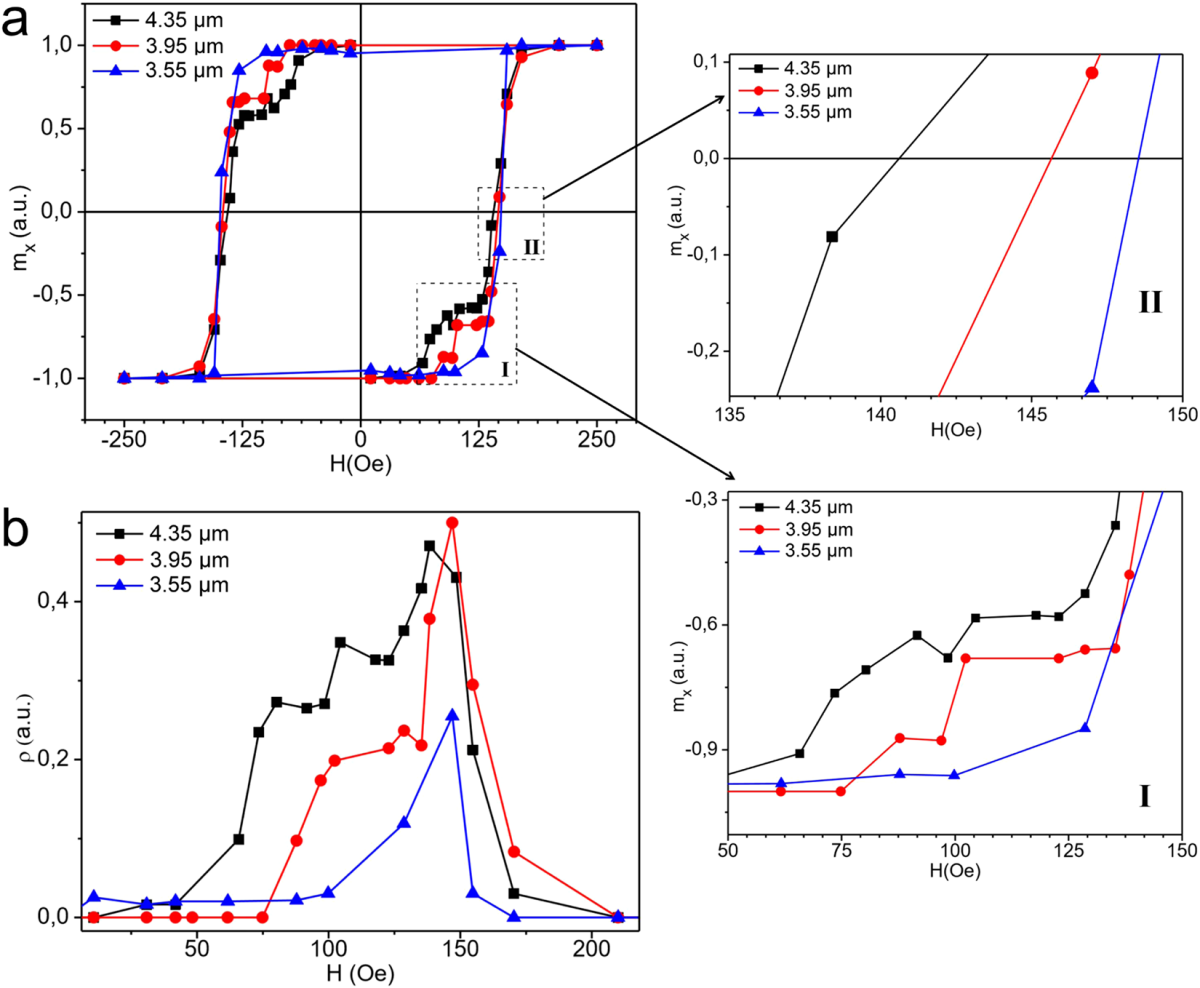
Experimental results for (**a**) the hysteresis trace and (**b**) magnetic charge density. The experiments were accomplished with three arrays of artificial spin ices having the same size 100*μ*^2^ but with different lattice parameters *a*. Black, blue and red lines represent compounds with *a* = 3550 nm, *a* = 3950 nm and *a* = 4350 nm respectively.

**Figure 4:**
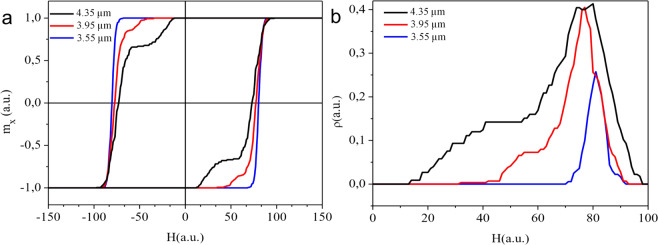
Theoretical calculations for the (**a**) hysteresis trace and (**b**) magnetic charge density for three arrays of artificial spin ices with the same size (100 *μ*^2^) but with different lattice parameters *a*. Again, black, blue and red lines represent compounds with *a* = 3550 nm, *a* = 3950 nm and *a* = 4350 nm respectively.

Before considering the theoretical results we still measure the hysteresis traces and the monopole density for three square ices with the same size (100*μ*^2^) but with different lattice parameters (*a* = 3550 nm, *a* = 3950 nm and *a* = 4350 nm). Such experiments are shown in Fig. 3. For the square ice with the smallest lattice spacing (*a* = 3550 nm), the cracking reversal effect practically does not occur and the monopole density exhibits a behavior very similar to the case discussed earlier, although with less presence of charges (blue lines in Fig. 3a,b). The smaller density *ρ* is due to a stronger coupling among the magnetic islands (as compared with the other cases), which implies in more difficulty to flip the spins in the beginning of the magnetization reversal process (on the other hand, after starting the spins flip, it is easier to create larger amounts of spin flip avalanches). As the parameter *a* increases, the cracking reversal becomes more evident (red and black lines for *a* = 3950 nm and *a* = 4350 nm respectively). In addition, the density of monopoles also tends to increase as *a* increases. Since these arrays are finite, the border effects on the propagation of monopoles play a fundamental role. These influences become larger as the lattice spacing increases. As the sample size is restricted by the experimental procedure, a square ice with small *a* has more nanoislands and behave as a bigger system, which should produce fewer edge effects. Therefore, one could perceive such border impacts as due to defects or vacancies of the spin ice lattice. It is exactly what we will do for applying the emergent model to compare theory and experiments. For completeness, Fig. 3 also shows the zoom of two regions (indicated by *I* and *II*) in the hysteresis loop, which can be directly compared with the simulation results (the lines highlighted in both regions are in the same sequence as their corresponding theoretical ones obtained by the simulations displayed in Fig. 4). However, we have opted for not exhibiting a zoom of other pertinent region, i.e., the zone around the magnetization saturation (where *H* ~ 160*Oe*). Indeed, this zone presents a fast transition, generating an intrinsic obstacle for getting reliable experimental data. Theoretical results demonstrate that the cracking reversal process also exist in this region. Nevertheless, it is much less pronounced than that distinctly observed in the hysteresis loop (Figs. 3a and 4a).

### Theory and Simulations

The emergent theory given by Hamiltonian *H*_*v*_ is now analyzed. Firstly, we would like to remark that, in the theoretical model considered in this work, modifications on the lattice spacing does not modify the Hamiltonian (*l* is given in units of the lattice spacing *a*). Thus, the experimental findings of the preceding section for systems with different lattice spacings are due to modifications in the monopole (*q*) and dipole (*m* = *ql*) values as well as on the field barrier for a spin flip. We begin our analysis by simulating the hysteresis trace. Following refs. ^11,26,27^, the magnetic field produced by the emergent excitations at each spin is summed to the external applied field and spin reversals occur when the total field at a given spin exceeds a certain threshold, i.e, if ${\overrightarrow{B}}_{i}^{tot}\cdot {\overrightarrow{s}}_{i} < -{h}_{i}$, where ${\overrightarrow{B}}_{i}^{tot}$ is the total field at spin ${\overrightarrow{s}}_{i}$ and *h*_*i*_ is a switching constant field with different values for different spins. When a spin is flipped, the adjacent sites have modifications in their emergent excitation. We must point out that the simulations (as well as the experiments) were performed in a quasi-static process, in the sense that, after each sufficiently small field step (0.1, in arbitrary units, in the simulations) the system has enough time to equilibrate before any measurement is done. After each field step, all spins are tested against the possibility to flip, i.e., the above condition for ${\overrightarrow{B}}_{i}^{tot}\cdot {\overrightarrow{s}}_{i}$ is verified and the spin is flipped if it is satisfied. After each flip, local fields are updated and the verification process starts over until there is no more spins left to be flipped for that field value. This may also lead to highly non equilibrium phenomena such as avalanches since many spins are flipped in a single field step in a stochastic process.

We first investigate a regular lattice containing magnetic charges in “pure” state, i.e., the difficulty to reverse a spin, *h*_*i*_, is drawn from a gaussian centered at *h*_*c*_ = 100 (in arbitrary units) with standard deviation *σ* = 5. Indirectly, it is somewhat equivalent to have the same distribution for the strength of charge |*q*| for the monopoles around the array, since the distribution of the reversal field may be mapped on the distribution of the charge strength |*q*|^11,26^. After that, the hysteresis trace is calculated following the method presented in ref. ^11^. Our simulation shows that, in this regime, the hysteresis trace does not contain significant cracking reversal effect and, therefore, the pure system displays an effective similarity with the experimental array having the smallest lattice parameter (*a* = 3550 *nm*). Indeed, the interpretation is that the spins are more inflexible for the small *a* parameter, since they have stronger interactions because the nanoislands are more concentrated in space. It implies in a smaller density of monopoles, reducing the problem of charges interaction, which is the main responsible for changes in the hysteresis loop as we will see below. Therefore, the array with small *a* should keep more resemblance with “pure” systems. However, in general, defects in magnetic systems (including nanomagnets) cause interesting behaviors in the compounds properties^9,28,29^. In particular, for *ASI* systems, defects such as vacancies or nanoislands imperfections^9^ may change the local strength of |*q*| in some vertices of actual lattices. We notice here that the |*q*| variation can be incorporated into the disorder of the spins reversal barriers as done by Budrikis *et al*.^26^ and Rodrigues and Mol^11^. Therefore, we introduce such defects in a percentage of nanoislands by modifying their difficulty of spin reversions, *h*_*i*_. Here, we have considered that 10% of the nanoislands have defects (randomly distributed around the array) in which the reversion field is altered in comparison with the remaining spins. In addition, the monopole strength |*q*| was reduced and the dipole moment *m* was increased as a consequence of the increase of the lattice spacing. Figure 4 shows the theoretical calculations for the hysteresis trace and monopole density for three cases where these impurities are introduced in the system: following the experimental data, in Fig. 4a, blue, red and black lines are associated to lattices with *a* = 3350 *nm*, 3950 *nm* and 4350 *nm* respectively. In Table 1 we summarize the simulation parameters used to reproduce the experimental findings for each lattice spacing. As can be seen, the charge strength was reduced and the dipole strength was increased. The reversal barrier follows a bimodal distribution, whose modes also decrease and distribution width increases.

**Table 1:** Parameters used in the simulations. ${h}_{c}^{90}$ stand for the mean reversal barrier for 90% of the spins and ${h}_{c}^{10}$ is the mean barrier for the remaining 10% of the spins. The standard deviation from these distributions, *σ*, is given as a percentage of *h*_*c*_.

*a*	|*q*|	*m*	${h}_{c}^{90}$	${h}_{c}^{10}$	*σ*
3550	1.60	0.45	100	100	5%
3950	1.45	0.48	95	70	7,5%
4350	1.40	0.50	90	40	10%

Then, except for the first case discussed above (blue curves), we notice that now the cracking reversal effect appears and becomes more pronounced as the lattice constant increases. The good qualitative agreement between the theoretical calculations and the experimental results is striking, demonstrating that, the inhomogeneity in the overall values of the magnetic charges and field barrier due to the presence of defects, are the main responsible for the cracking reversal outcome in the hysteresis trace. Really, from the experimental point of view, in our nearly accurate arrays, the edge deficit spins must be the key ingredient for interpreting the defects added in the theoretical calculations. Indeed, we may expect that these edges spins have reversion barrier energy different from the bulk spins. As shown in ref. ^30^, the spins in the *ASI* borders are more arduous to move when the external magnetic field is applied in their direction. Therefore, lattices with larger constant *a* must present less impediment to have processes of edge spin reversals, implying in larger quantities of charges near the borders. These are then the defects in our experimental case. As a consequence, such lattices also contain larger density of monopoles (see Fig. 4b). In our experimental investigation, all arrays have the same size (100 *μm*^2^) but different lattice constants. Note that the array with larger lattice constant (*a* = 4350 *nm*) has about 10% of its spins (~40 spins) located exactly at the left and right borders (with nanoislands in the horizontal position having spins pointing along the magnetic field direction). Thus, this array is apparently closer to the theoretical calculation conditions, while the other two arrays, even containing a bigger number of spins in the left and right borders on the one hand, would have a much higher spin reversal barrier energy on the other hand. It is a strong obstacle for rising monopoles at their edges. Nevertheless, there are fewer Coulomb interactions (in overall) among normal charges and “charges with defects”, leading to a smaller cracking reversal in the hysteresis loop. We remark that the Coulomb interaction is the main term in Hamiltonian given by *H*_*v*_.

## Conclusion

We have experimentally and theoretically investigated square *ASI* arrays directly by means of their vertices excitations (monopole quasiparticles and dipoles) using an emergent model. Theoretical calculations agree qualitatively and in some cases quantitatively with experimental results, permitting us to identify clearly the roles of magnetic charges in the properties of the system. In particular, defects in monopole like excitations seems to be the key ingredients responsible for the presence of a cracking reversal in the hysteresis curve. Indeed, such defects modify the charges and, consequently, the Coulombian interaction among the monopoles; in our model, these modifications are equivalent to modifications in the reversal field barrier of the nanoislands. In our system, these defects are more relevant in the spin deficit at the edges of *ASI* than the inhomogeneities in the magnetic nanoislands that naturally occur in the fabrication process. These results may impact the development of devices based on the magnetic field driven dynamics of monopoles in ASI, possibly allowing the development of island geometries that enhance the monopole charge in some area of the lattice, modifying the entire dynamic response of the system. In addition, our results give more consistence for the real presence of Nambu magnetic monopoles^9,10^ in artificial spin ices, since the calculations are based essentially on the Coulomb interaction.
